# Tailoring the Properties of Marine-Based Alginate Hydrogels: A Comparison of Enzymatic (HRP) and Visible-Light (SPS/Ruth)-Induced Gelation

**DOI:** 10.3390/md24010022

**Published:** 2026-01-02

**Authors:** Feiyang Wang, Emmanuelle Lainé, Paolina Lukova, Plamen Katsarov, Cédric Delattre

**Affiliations:** 1 Université Clermont Auvergne, Clermont Auvergne INP, CNRS, Institut Pascal, 63000 Clermont-Ferrand, France; feiyang.wang@doctorant.uca.fr (F.W.); cedric.delattre@uca.fr (C.D.); 2 UMR454 MEDIS, INRAE-UCA, Université Clermont Auvergne, 63000 Clermont Ferrand, France; emmanuelle.laine@uca.fr; 3 Department of Pharmacognosy and Pharmaceutical Chemistry, Faculty of Pharmacy, Medical University of Plovdiv, 4002 Plovdiv, Bulgaria; 4 Department of Pharmaceutical Technology and Biopharmacy, Faculty of Pharmacy, Medical University of Plovdiv, Vasil Aprilov Str. 15A, 4002 Plovdiv, Bulgaria; plamen.katsarov@mu-plovdiv.bg; 5 Research Institute at Medical University of Plovdiv, Vasil Aprilov Str. 15A, 4002 Plovdiv, Bulgaria; 6 Institut Universitaire de France (IUF), 1 Rue Descartes, 75005 Paris, France

**Keywords:** phenol functionalization, 3D bioprinting, tissue engineering, photo crosslink

## Abstract

Alginate is a natural polysaccharide extracted from brown algae and is commonly used as a biomaterial scaffold in tissue engineering. In this study, we performed phenol functionalization of sodium alginate based on chemical modification methods using 1-ethyl-(3-dimethylaminopropyl)carbodiimide/N-hydroxybutanediimide/2-(N-morpholino) ethanesulfonic acid (EDC/NHS/MES) and tyramine. The presence of phenol groups was confirmed by spectrophotometry and Fourier Transform Infrared. We successfully prepared hydrogels using a horseradish peroxidase/hydrogen peroxide (HRP/H_2_O_2_) enzymatic system as well as an sodium persulfate (SPS)/ruthenium light-crosslinking system. Optimization identified 1 mM ruthenium and 4 mM SPS as the most effective photo crosslinking conditions. At the same time, 1 mM H_2_O_2_ and 10 U/mL HRP are considered optimal conditions for the enzyme-linked reaction. Rheological measurements monitored the gelation process, revealing that the viscosity, storage modulus, and loss modulus of the material increased by at least one hundredfold after crosslinking. Thixotropy results demonstrated excellent recovery of the material. Texture analysis indicated that the crosslinked material possessed notable strength and toughness, highlighting its potential applications in tissue engineering after 3D bioprinting.

## 1. Introduction

Natural polysaccharides (e.g., alginate, xanthan, and cellulose) have been widely used in the production of inks in 3D printing as biomaterials for natural polymers. Among them, alginate hydrogels are widely used for the controlled and localized delivery of drugs and biomolecules in tissue regeneration. Traditional drug delivery methods, such as oral tablets or subcutaneous injections, often cause sharp increases in drug concentration throughout the body and fail to target the desired tissues. This imbalance can lead to strong side effects and reduced treatment efficiency [1]. These methods also usually fail to maintain a steady drug level over time [2]. To address this problem, researchers often turn to polymer-based carriers that release small, steady amounts of the drug at the target site. This controlled release approach helps maintain a high local drug concentration for longer periods while minimizing unwanted effects on surrounding tissues [3]. Meanwhile, alginate-based biomaterials have been widely used in tissue repair and regeneration. With the rapid development of bioprinting technology, alginate has become the preferred bio-ink for constructing various tissue substitutes [4]. Bioinks are typically defined as material formulations that contain living cells and can be used with automated biomanufacturing technologies [5]. In both extrusion and lithography-based bioprinting, bioinks and bioresins are usually made as water-swollen polymer networks, also known as hydrogels. Hydrogels are attractive because they offer a wide range of tunable chemical, mechanical, and biological properties, and they can support cell encapsulation. These hydrogels can be formed through several methods, including enzymatic reactions [6], redox processes [7], and external triggers like temperature or light [8]. Among these, photocrosslinking stands out as especially useful for 3D printing and additive manufacturing. This is because light-based crosslinking offers built-in spatial and temporal control, which helps fine-tune the printing process and build complex 3D structures with precision. Alginate 3D bioprinting has also found widespread application in cancer treatment. Currently, alginate models for various cancer types, including breast cancer, liver cancer, and colorectal cancer, have been obtained using 3D bioprinting technology. These models exhibit higher physiological relevance by improving cell tissue, gene expression profiles, and drug response characteristics [9].

Carbodiimide chemistry, especially using 1-ethyl-(3-dimethylaminopropyl)carbodiimide and N-hydroxybutanediimide (EDC/NHS), is widely used in polymer synthesis [10]. The process starts when EDC activates carboxyl groups to form an unstable O-acylisourea intermediate. This intermediate is then attacked by an amine group (–NH_2_), forming a stable amide bond. NHS helps by converting the intermediate into a more stable ester, which improves the overall efficiency and stability of the reaction. Since both amino and carboxyl groups are commonly found in polysaccharides and in catechol or pyrogallol structures, using EDC/NHS chemistry to modify polysaccharides with catechol or pyrogallol is a practical and promising strategy. Carbodiimide-mediated reactions can be effectively used to couple polysaccharides containing carboxyl groups—such as hyaluronic acid (HA), alginate, or oxidized cellulose—with small molecules bearing amino groups, including catechols and pyrogallols (e.g., dopamine, 5-hydroxydopamine). For example, purified carboxymethylated bacterial cellulose (CM-BC) can be dissolved in phosphate-buffered saline (PBS, pH 6.0) under vigorous stirring. EDC and NHS are then added to activate the carboxylic acid groups of CM-BC. Subsequently, an aqueous solution of dopamine is introduced, and the reaction is allowed to proceed under an inert atmosphere for 24 h. This results in the partial functionalization of bacterial cellulose with catechol groups [11]. In addition, carbodiimide chemistry can also be applied to reactions between amino-containing polysaccharides—such as chitosan and its derivatives—and carboxylic acid-containing catechol molecules, including caffeic acid and gallic acid (GA). For instance, Huang et al. developed a catechol-conjugated chitosan (CHI-C) hydrogel exhibiting multifunctional properties such as tissue adhesion, self-healing, cytocompatibility, hemocompatibility, and blood cell coagulation. The hydrogel was synthesized by grafting caffeic acid onto the chitosan backbone via EDC/NHS-mediated carbodiimide coupling, achieving a grafting efficiency of approximately 11.3% [12]. In the carbodiimide coupling method, the carbodiimide reagent itself does not become part of the final crosslinked structure but serves as a facilitator for bond formation [13]. This reaction is advantageous due to its mild operating conditions, minimal by-product formation, and good biocompatibility [14]. However, the method is often limited by unavoidable side reactions—such as the formation of surface-grafted anhydride products from adjacent carboxyl groups or N-acylureas via intramolecular acyl transfer. These competing reactions can lead to relatively low coupling efficiencies, typically yielding grafting rates of around 10%, which in turn may compromise the adhesive performance of the resulting material [15]. Research on phenolized alginate has become a hot topic in recent years. A study reported a phenol-grafted alginate hydrogel (AlgS-Ph) and explored its application in FGF-2 delivery. This hydrogel was prepared via a horseradish peroxidase (HRP)-mediated crosslinking reaction. The hydrogel did not show cytotoxicity against 10T1/2 cells. Angiogenesis experiments showed that FGF-2 released from the AlgS-Ph hydrogel promoted angiogenesis [16].

Tyramine (Tyr) is a naturally occurring biogenic amine that forms when bacteria decarboxylate the amino acid tyrosine. It is commonly found in foods, animals, plants, and microorganisms [17]. In recent years, tyramine has often been used to modify biopolymers like polysaccharides and peptides. This is typically achieved through amination, where tyramide reacts with carboxyl groups on the biopolymer backbone [18]. Tyramine is used because it introduces phenol groups that help achieve fast, controlled in situ gelation. This makes it easier to design hydrogel hybrids with specific bioactive or mechanical properties [19]. One widely studied example is hyaluronic acid modified with tyramine (HA-Tyr). These hydrogels have shown great potential for the sustained delivery of proteins and anti-cancer drugs. They are also used in cartilage replacement, thanks to their ability to interact with tyrosine residues in the cell’s surrounding matrix. Since tyramine induces multiple interactions between different types of polysaccharides, hybrid hydrogels based on Pectin-Tyr mixed with other cationic polysaccharides can promote gelling, mechanical properties, and cellular functions, making them more practical for complex scenarios such as 3D bioprinting [20]. Because this method is both simple and efficient, it has been widely used to the other polysaccharides as well. Examples include alginate, dextran, and carboxymethylcellulose (CMC) modified with tyramine (CMC-Ph) [21].

Horseradish peroxidase (HRP) is an enzyme widely used to initiate polymer crosslinking reactions. When introduced into a polymer–phenol solution, HRP reacts with hydrogen peroxide (H_2_O_2_) to catalyze the formation of covalent bonds between phenol groups, resulting in a stable crosslinked network [22]. The gelation time of a hydrogel plays a crucial role in determining its final physicochemical and mechanical properties. This enzymatic reaction typically takes place under mild conditions, such as neutral pH and room temperature, making it ideal for tissue engineering applications. The duration of gelation is mainly influenced by the concentration of HRP, while the amount of H_2_O_2_ directly affects the stiffness and overall mechanical strength of the gel. HRP-crosslinked gelatin hydrogels enriched with type I collagen have been shown to promote various cellular behaviors, including strong cell adhesion and the chondrogenic differentiation of human mesenchymal stem cells (MSCs) [23].

Light-driven redox reactions are often used to photocrosslink bioinks. In these systems, polymers that contain phenolic groups can form crosslinked networks through photooxidation followed by radical coupling between active groups [24]. This process requires photosensitizers—special dyes or additives that absorb light and move into an excited state. Once excited, these photosensitizers can oxidize the target functional groups in the polymer. For a photosensitizer to work effectively, it needs to meet several key requirements. It should have a high absorption rate, a strong quantum yield, and enough chemical stability to drive photooxidation under the chosen light conditions [25]. When these criteria are met, the photosensitizer can generate reactive radicals either by transferring electrons or by pulling hydrogen atoms from nearby molecules. Polysaccharides that carry functional groups like carboxyl or phenol can also be physically crosslinked to improve the printability and stability of bioinks after extrusion. Physical crosslinking involves reversible, non-covalent interactions that help form a polymer network. This includes mechanisms like peptide self-assembly, ionic bonding, and supramolecular interactions [26]. One common example is alginate, which can be crosslinked with Ca^2+^ ions. This method supports the production of degradable hydrogels for extrusion-based printing [27]. Studies have also shown that alginate can serve as a temporary, or “sacrificial,” material to make low-viscosity polymers easier to print. In a coaxial extrusion setup, a mixture of alginate and gelatin is pushed through an inner needle, while calcium chloride flows through the outer needle. At the tip, alginate immediately crosslinks in the presence of calcium, forming a deposited structure. After printing, the gelatin is stabilized through photocrosslinking, and the ionically crosslinked alginate is then removed by washing [28]. Although photocrosslinking is widely used in 3D bioprinting, the conditions under which it is carried out—such as light source, intensity, exposure time, wavelength, and photoinitiator concentration—can vary greatly between labs. This variation makes it hard to compare results across different studies, even when the same bioink and photoinitiator are used [29]. In recent years, one of the most notable radical photoinitiator systems is tris(2,2-bipyridine)dichlororuthenium(II) hexahydrate (Ru). This system is based on a transition metal complex. Ruthenium strongly absorbs visible light, with a molar extinction coefficient of 14,600 M^−1^·cm^−1^ at 450 nm [30]. When exposed to visible light, the ground-state Ru^2+^ becomes excited. It then donates an electron to a co-initiator, such as sodium persulfate (SPS), and is oxidized to Ru^3+^. After receiving the electron, SPS breaks down into sulfate anions and sulfate radicals. These radicals can then initiate free radical chain polymerization or thiol-ene photocrosslinking. Interestingly, the photoexcited Ru^3+^ can also drive light-triggered redox reactions by oxidizing phenolic groups like tyrosine [31]. These oxidized tyrosine groups are then converted into tyrosyl radicals, which can react with other tyrosyl radicals nearby to form dityrosine bonds [32].

In the early development of biomanufacturing and bioink materials, many bioprinting techniques began using light. These techniques generally fall into two categories: extrusion-based bioprinting and photolithography-based bioprinting. Extrusion methods use bioinks that can be crosslinked with light, while photolithography methods rely on light to turn resins or polysaccharides into solid structures. Light offers precise control over where and when the material reacts, making it easier to form complex 3D shapes. Light-based reactions are also very efficient and usually produce very few by-products. This is important for building structures that contain living cells. Because of this, researchers have put a lot of effort into designing and synthesizing photocrosslinkable polymer bioinks and bioresins. These materials need to be biocompatible, bioactive, and degradable to support and enhance the creation of functional 3D tissue structures. Choosing the right photoinitiator is critical for most light-based bioprinting methods. The efficiency and reactivity of the photoinitiator directly affect how much light and how long it needs to be applied. For example, increasing the concentration of Irgacure 2959 from 0.05% to 0.5%, or raising the light intensity from 3 mW/cm^2^ to 100 mW/cm^2^, caused a major drop in cell viability [30]. This issue was improved by switching to the Ru/SPS system, which uses visible light instead of UV. Likewise, a study by Colosi et al. [28] showed that longer UV exposure times also lowered cell viability.

Many of the advances and visual breakthroughs in photocrosslinking-based bioprinting come from using flexible and customizable photocrosslinking techniques. These methods make it possible to design hydrogel bioinks that work well with different bioprinting and manufacturing approaches—especially extrusion-based printing using shear-thinning materials. The most important factor in using these techniques is choosing the right combination of materials. This includes the base polymers, photoinitiators, and how they interact with cells. It is also essential to select light sources and light intensities that are compatible with the system and safe for cells. In this study, we synthesized two hydrogels through chemical modifications involving tyramine-mediated phenol functionalization. Successful phenolization of the polysaccharides was confirmed by quantifying the phenol content. We then attempted gelation of the modified polysaccharides using the Ru/SPS photoinitiating and HRP/H_2_O_2_ system, optimizing both the photoinitiator and enzyme concentration and crosslinking duration. The parameters of the light source were also adjusted and standardized accordingly. Rheological analysis was performed to evaluate the viscosity of the hydrogels before and after crosslinking. Additionally, the viscoelastic properties and printability of the Alg-Ph formulations were systematically compared. The aim of this study is to use two hydrogels, prepared by enzymatic crosslinking and photocrosslinking of phenol-modified alginate, as inks for 3D printing, ultimately for tissue engineering.

## 2. Result and Discussion

### 2.1. UV-Absorbance of the Alginate Derivative

**Figure 1:**
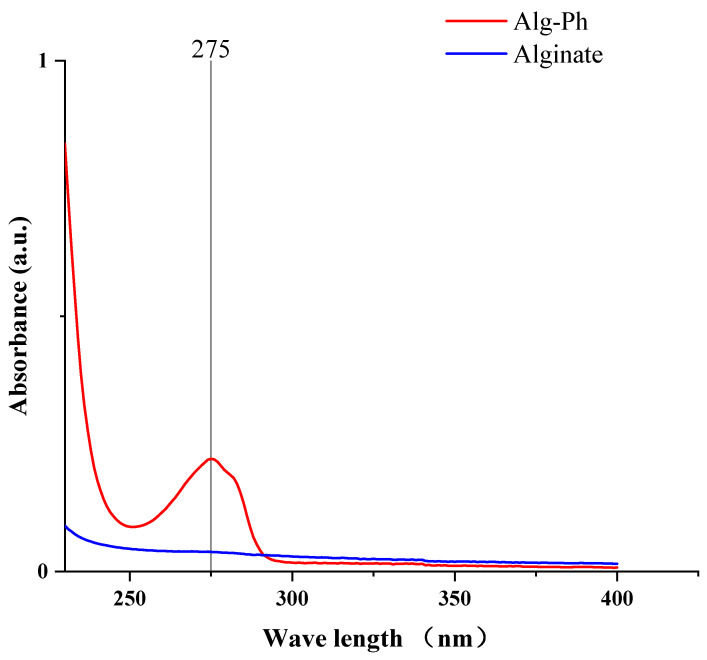
UV-absorbance of alginate and Alg-Ph.

Tyramine was used as the grafting agent to add phenol groups. After grafting, Alg-Ph was washed one time of acetone and ten times to remove free tyramine. The washing process was considered complete when the absorbance of the rinse solution at 275 nm was less than 0.01. UV absorbance measurements of the final 0.1% phenolized solutions (Figure 1) showed that the degrees of phenol grafting was 2.1%.

### 2.2. Fourier-Transform Spectroscopy of the Alginate-Derivative

**Figure 2:**
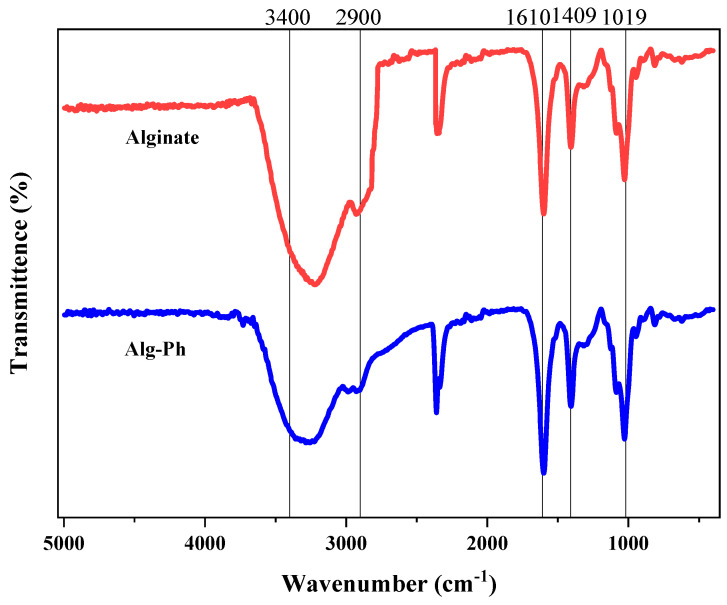
FTIR spectra of Alginate and Alg-Ph.

FTIR was used to identify the functional groups present in the samples, as shown in Figure 2. The broad peak at 3412 cm^−1^ is attributed to the O–H stretching vibration of hydroxyl groups [33]. The peak at 2900 cm^−1^ corresponds to C–H stretching vibrations [34]. A key peak at 1642 cm^−1^ indicates the C=O stretching vibration of carboxyl groups [35]. The peak at 1000 cm^−1^ is associated with C–H stretching in glycosidic bonds [36]. Additionally, the peak near 600 cm^−1^ is related to the C–O stretching vibration characteristic of alginate [37]. The FTIR results reveal changes in functional groups following chemical modification of alginate. These findings confirm the successful introduction of carboxyl groups to phenolic groups through phenolization. Although alginate and Alg-Ph share glucose as their basic structural unit, their distinct molecular structures result in notable differences in functional group content, bonding patterns, and modification efficiency.

### 2.3. Gelation Process Optimization

**Figure 3:**
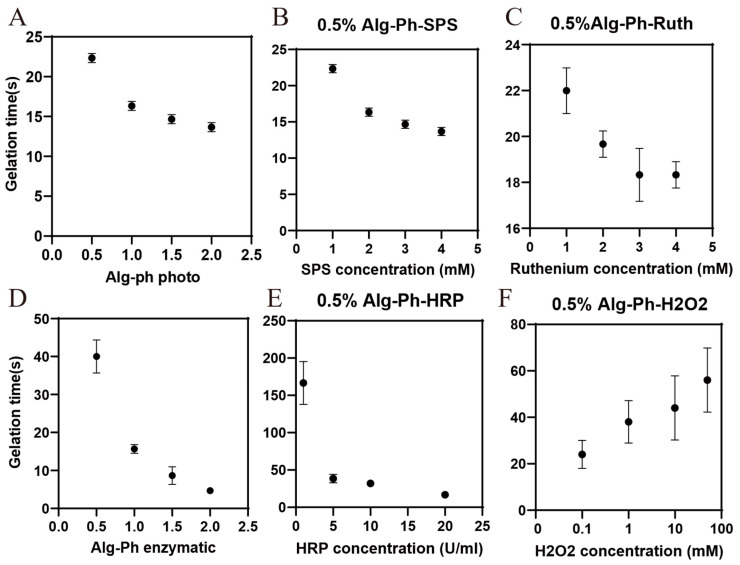
Optimization of gelation time for photocrosslinking of (**A**) 0.5–2% Alg-Ph at (**B**) 1–4 mM SPS and (**C**) 1–4 mM ruthenium. Enzymatic optimization of (**D**) 0.5–2% Alg-Ph at (**E**) 5–20 mM ruthenium and (**F**) 0.1–50 mM H_2_O_2_.

During the optimization of the photoreticulation (Figure 3A) and enzymatic crosslink system (Figure 3B), we observed that increasing the concentration of the polymer reduced the crosslinking time. However, even at low concentrations, the viscosity of the solution remained high, which can limit practical use. To balance these factors, we selected the lowest crosslinkable concentrations of alginate derivative for further optimization. We also found that higher concentrations of sodium persulfate (SPS) led to faster crosslinking and harder hydrogels. The results showed that the crosslinking time decreased with increasing SPS levels. SPS, when activated by ruthenium, produces sulfate radicals (SO_4_^−^·), which initiate the oxidation of tyrosine groups to form dityrosine bonds. These bonds improve the mechanical strength and elasticity of the resulting hydrogel [29]. Previous research has shown that, in casein hydrogels, optimizing SPS at 4 mM and ruthenium at 1 mM produced hydrogels with adhesion strength sufficient to withstand over 180 mmHg of blood pressure, suggesting strong clinical potential [38]. In optimizing ruthenium levels, we noticed that higher ruthenium concentrations increased crosslinking time. Since ruthenium can be toxic, and the literature commonly uses it at concentrations between 0.5 mM and 4 mM, we recommend using 1 mM for practical and safe photopolymerization [39]. Similarly, for enzyme crosslinking, we found that higher Alg-Ph concentrations resulted in shorter crosslinking times. We optimized HRP at different concentrations using 0.5% Alg-Ph and found that the crosslinking times were not significantly different when the enzyme concentration ranged from 5 to 20 U/mL. Then, we set the enzyme concentration to 10 U/mL (because 10 U/mL exhibited high viscosity in subsequent viscosity analysis) and tested different concentrations (0.1–50 mM) of H_2_O_2_ with 0.5% Alg-Ph. The results showed that the crosslinking time increased with increasing H_2_O_2_ concentration. Therefore, we recommend using 10 U/mL HRP and 0.1 mM H_2_O_2_ as the enzyme crosslinking system.

### 2.4. Analysis of Hydrogel Properties

**Figure 4:**
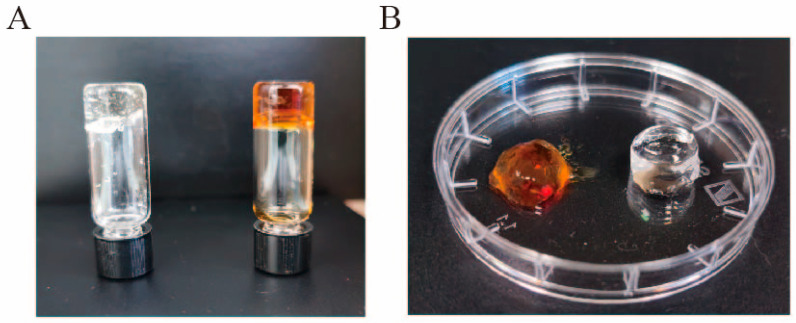
Crosslinking analysis after photocrosslink: (**A**) Inverted bottle method in the left is 500 μL Alg-Ph with 0.1 mM H_2_O_2_ and 10 U/mL HRP, in the right is 500 μL Alg-Ph with 1 mM ruthenium and 4 mM SPS. (**B**) Exhibition of hydrogel in Petri dish in the left is 500 μL Alg-Ph with 1 mM ruthenium and 4 mM SPS, in the right is 500 μL Alg-Ph with 1 mM H_2_O_2_ and 10 U/mL HRP.

After optimizing the system, we tested photoreticulation and enzymatic crosslinking (Figure 4) of hydrogels. We used the inverted vial method (Figure 4A) to demonstrate the successfully prepared hydrogel. When it changes from a liquid to a solid state, rotating the vial causes it to settle at the bottom. This showed that both crosslinking methods were successful. We tested crosslinking systems of 500 μL mainly because we often need smaller systems when performing cell survival and cytotoxicity experiments (Figure 4B). Droplet-shaped hydrogels have higher requirements for cell adhesion, and smaller hydrogels are more convenient for observing the state of cells. Of course, the comparison between small and large volumes shows that volume is one of the important factors affecting the crosslinking time of hydrogels. However, crosslinking can be completed at the same concentration regardless of the volume, likely due to improved light penetration exposure time required.

**Figure 5:**
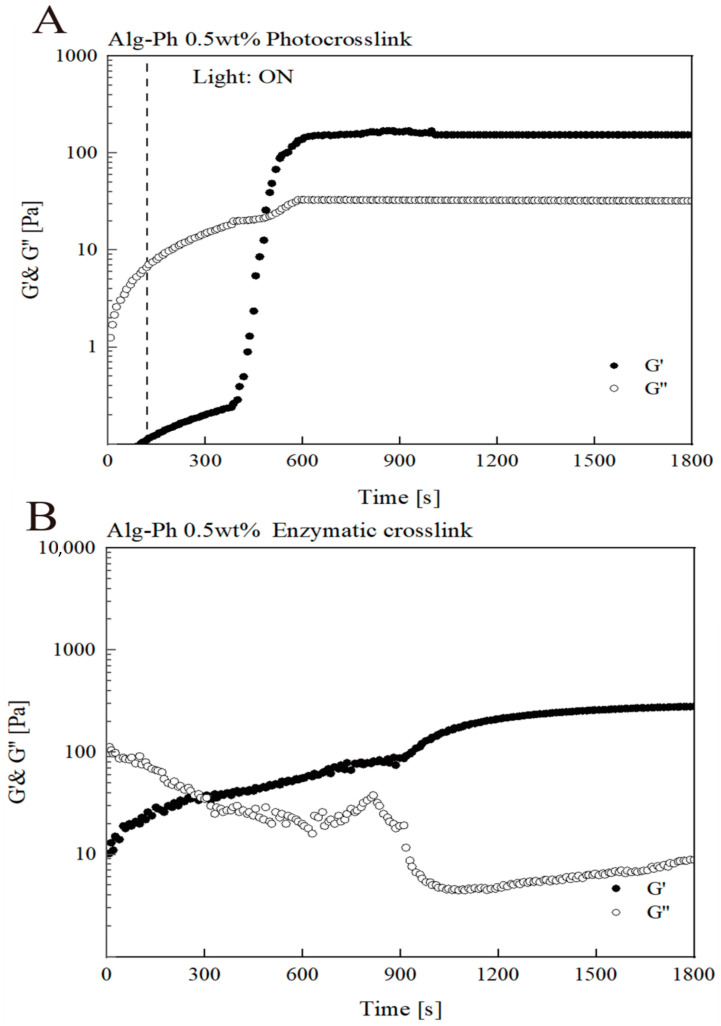
Longtime crosslinking analysis. (**A**) Changes in G′ and G′′ over time under visible-light exposure (4 W/m^2^ @ 452 nm) for Alg-Ph containing 44.0 mM SPS and 1.0 mMRu(bpy)s under 2% strain at 1 kHz frequency. (**B**) Changes in G′ and G′′ over time during enzymatic crosslinking for Alg-Ph containing 0.1 mM H_2_O_2_ and 10 U/mL HRP under 2% strain at 1 kHz frequency.

To monitor the transition from liquid to gel, we performed long-term rheological analysis (Figure 5). For the first two minutes, measurements were taken in the dark. At the two-minute mark, light exposure began. For fast-crosslinking hydrogels of Alg-Ph (Figure 5A), crosslinking completed in about 5 min, as indicated by the stabilization of G′ (storage modulus) and G′′ (loss modulus), with G′ surpassing G′′. It is important to note that the limited light exposure during rheological testing may have extended the observed times, making these values relative rather than absolute [40,41]. In the enzyme crosslinking test, we noticed that its gel point also appeared within 5 min. Simultaneously, it exhibited a stable solid form after crosslinking (Figure 5B).

**Figure 6:**
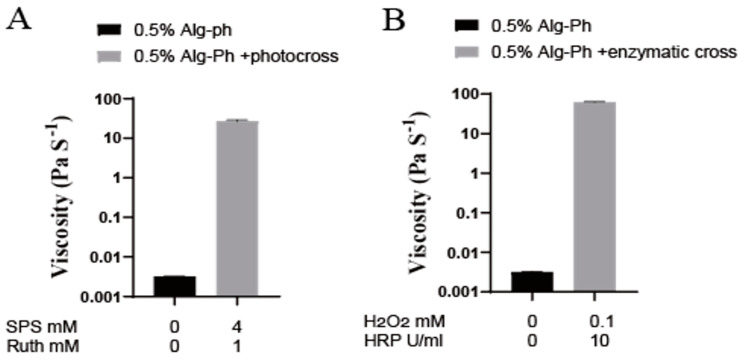
Viscosity of 0.5% Alg-Ph before and after photocrosslinking. (**A**) Photocrosslinking with 1 mM ruthenium and 4 mM SPS, (**B**) enzymatic crosslinking with 10 U/mL HRP and 0.1 mM H_2_O_2_.

**Figure 7:**
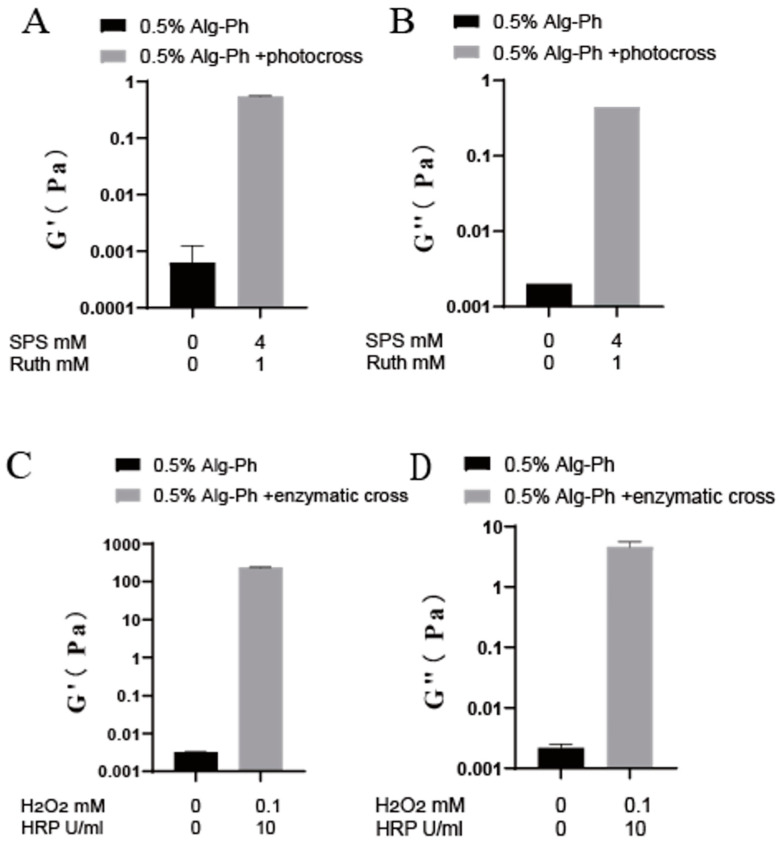
G′ and G′′ of 0.5% Alg-Ph before and after crosslinking. (**A**,**B**) Photocrosslinking with 1 mM ruthenium and 4 mM SPS, (**C**,**D**) enzymatic crosslinking with 10 U/mL HRP and 0.1 mM H_2_O_2_.

We then compared changes in viscosity, storage modulus, and loss modulus before and after crosslinking (Figure 6 and Figure 7). Viscosity increased by at least 1000 times of magnitude after crosslinking. We also observed that the viscosity increased substantially after crosslinking—from 0.003 Pa·s before crosslinking to 27.41 Pa·s after photocrosslinking and 62.47 Pa·s after enzyme crosslinking—indicating that enzyme-mediated crosslinking produced a greater increase in viscosity than photocrosslinking.

We compared the changes in storage modulus (G′) and loss modulus (G′′) before and after crosslinking. Both G′ and G′′ increased, but G′ increased by about one order of magnitude more than G′′, showing a clear transition from a liquid-like state (G′ < G′′) to a solid-like state (G′ > G′′)—a typical signature of gel formation [42].

**Figure 8:**
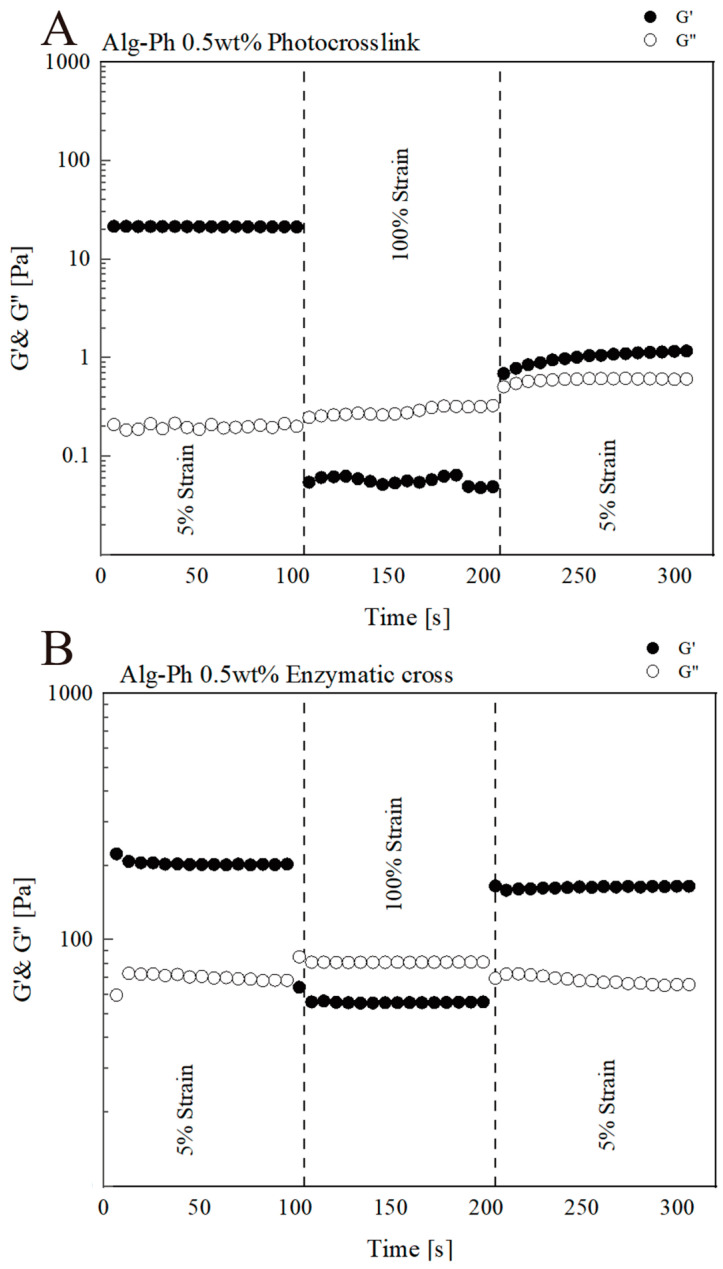
Rheological behaviors of 0.5% Alg-Ph. (**A**) Photocrosslinking and (**B**) enzymatic crosslinking under varied strain (5–100%) at 1 kHz frequency. Storage modulus: G′. Loss modulus: G′′.

Given the end goal of using these hydrogels in tissue engineering, we conducted a quantitative analysis of their thixotropic recovery behavior (Figure 8). Thixotropy reflects a material’s ability to recover after deformation and is crucial for bioinks used in 3D printing [43]. We used a three-step test: resting → high shear → recovery. Under high shear (100% strain), both G′ and G′′ dropped, with G′ falling below G′′, indicating the hydrogel became more fluid. After resting, the gel only partially recovered. G′ remained lower and G′′ remained higher than the original resting state, suggesting a slight loss in structural integrity after stress. This is typical of thixotropic materials, which show reversible but not fully complete recovery in mechanical strength.

### 2.5. Texture-Related Properties

To better understand the texture-related properties of the hydrogels—such as stiffness, elasticity, and cohesiveness—we performed a texture profile analysis. By combining rheological data (G′/G″ modulus) with mechanical texture metrics (such as hardness and resilience), we aimed to clarify how molecular structure influences the formation and stability of the hydrogel network structure [44,45].

**Figure 9:**
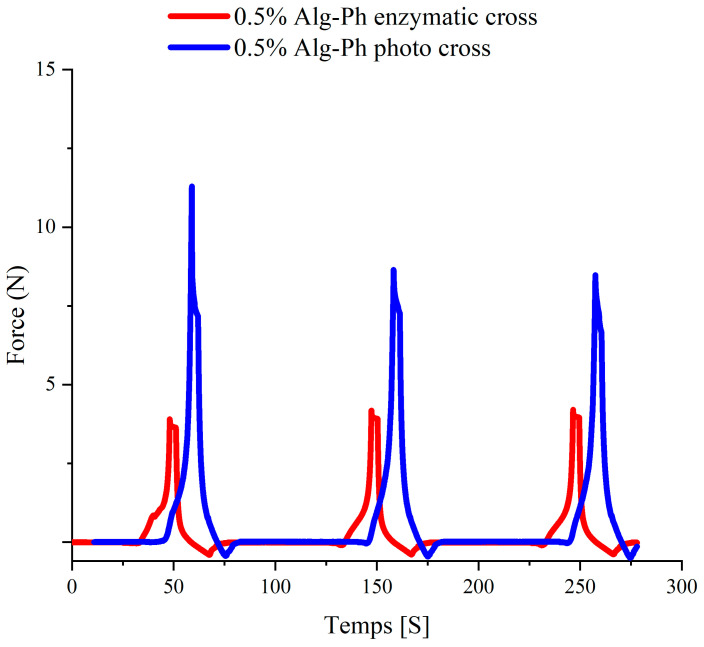
Texture analysis of 0.5% Alg-Ph photocrosslinking versus enzymatic crosslinking. Blue line: 0.5–2% Alg-Ph at 1–4 mM ruthenium and 1–4 mM SPS. Red line: 0.5–2% Alg-Ph at 0.1–50 mM H_2_O_2_ and 10 U/m HRP.

We began by analyzing the minimum crosslinking concentrations of four hydrogel materials through texture analysis (Figure 9). It was observed that the hardness of two hydrogels decreased across three consecutive testing cycles. This decline reflects energy loss due to elastic deformation and relates to the material’s ability to recover after compression—an important property for soft tissue mimics. Alg-Ph hydrogel showed multiple fracture peaks during testing, suggesting a higher risk of mechanical failure under stress. Additionally, we noted a positive correlation between viscosity (represented by peak area) and hardness, which is relevant in tissue engineering where stronger adhesion can support better cell attachment and integration [46].

**Figure 10:**
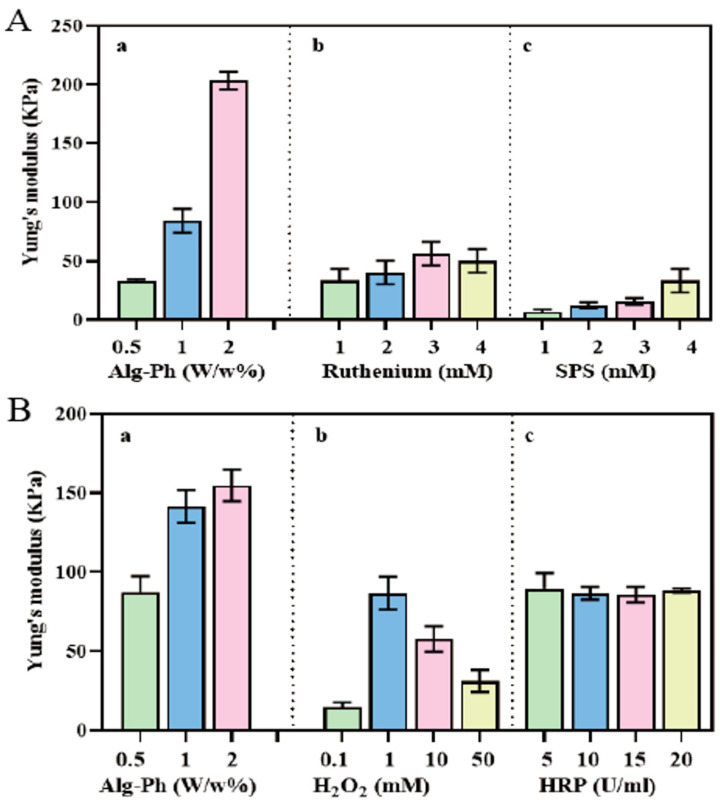
Dependence of Young’s modulus of hydrogels for concentrations of (**A**) 0.5–2% Alg-Ph (a) at 1–4 mM ruthenium (b) and 1–4 mM SPS (c), (**B**) 0.5–2% Alg-Ph (a) at 0.1–50 mM H_2_O_2_ (b) and 10 U/m HRP (c).

As shown in Figure 10, hydrogel stiffness increased with the concentration of Alg-Ph under fixed photoinitiator conditions (1 mM ruthenium and 4 mM SPS). For instance, the Young’s modulus of Alg-Ph photocrosslinking increased from 33 kPa at 0.5% (*w*/*v*) to 204 kPa at 2.0% (*w*/*v*), a 6-fold increase. These findings show that stiffness is positively correlated with polymer concentration. We also explored how photoinitiator concentrations affect stiffness. In general, hydrogel stiffness increased with ruthenium concentration, especially between 1 mM and 3 mM, although the change was less pronounced between 1 and 2 mM and 3 and 4 mM. For SPS, we observed a consistent upward trend in stiffness with increasing concentrations. For example, when SPS was increased from 1 mM to 4 mM, the Young’s modulus of Alg-Ph rose by about 6 times (Figure 10A). The Young’s modulus of Alg-Ph enzymatic crosslinking increased from 89 kPa at 0.5% (*w*/*v*) to 160 kPa at 2.0% (*w*/*v*), a 2-fold increase. We also explored how enzyme and H_2_O_2_ concentrations affect stiffness. In general, hydrogel stiffness did not change with HRP concentration. For H_2_O_2_, we observed that the Young’s modulus showed a trend of increasing initially and then decreasing as the concentration of H_2_O_2_ increased (Figure 10B).

### 2.6. Printability Analysis

**Figure 11:**
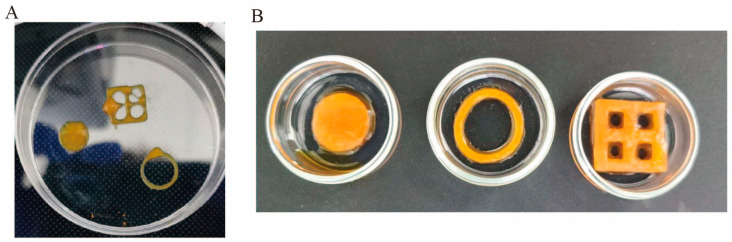
(**A**) Three-dimensional bioprinting of 0.5% Alg-Ph after photocrosslinking with 1 mM ruthenium and 4 mM SPS (for 3 different molds: lattice 2 × 2, ring and disk). (**B**) Silicone mold fabrication of 0.5% Alg-Ph after photocrosslinking with 1 mM ruthenium and 4 mM SPS (3 different molds: kept in 4 °C in silicone molds for 2 h and exposed under blue light for 5 min after removing the scaffolds).

Based on these optimizations, we carried out 3D extrusion-based printing using Alg-Ph (Figure 11A). We found that 2% Alg-Ph had suitable rheological properties for extrusion printing [47]. Aside from extrusion printing, we also tested scaffold fabrication using silicone molds (Figure 11B). While this method has limitations, it is simple, low-cost, and easy to implement. We injected the hydrogel solutions into silicone molds, allowed them to rest at 4 °C for two hours, and then exposed them to visible light for five minutes. This process resulted in three complete scaffold structures. We measured the Young’s modulus of these molded scaffolds using 2% Alg-Ph. All three scaffolds showed the same stiffness (Figure S1), indicating that the shape of the mold does not significantly affect the material’s mechanical strength. This result also confirms the robustness and flexibility of our chemically modified alginate-based hydrogels, which support multiple strategies for scaffold formation in tissue engineering applications.

## 3. Materials and Methods

### 3.1. Materials

In this experiment, Alginate (W201502—1KG) was obtained from Sigma-Aldrich^®^ (St Quentin Fallavier Cedex, France) [48]. Tyramine hydrochloride, N-hydroxysuccinimide (NHS), MES, H_2_O_2_ aqueous solution (33 *w*/*w*%), and water-soluble carbodiimide (WSCD) were purchased from Sigma-Aldrich (France). HRP was form Fujifilm Wako Pure chemical industries (Osaka, Japan). Dulbecco’s phosphate-buffered saline (1X) was procured from Sigma Aldrich (France). The syringe and the noddle for 3D printing were purchased form CELLLINK (Gothenburg, Sweden).

### 3.2. Chemical Modification of Alginate

**Figure 12:**
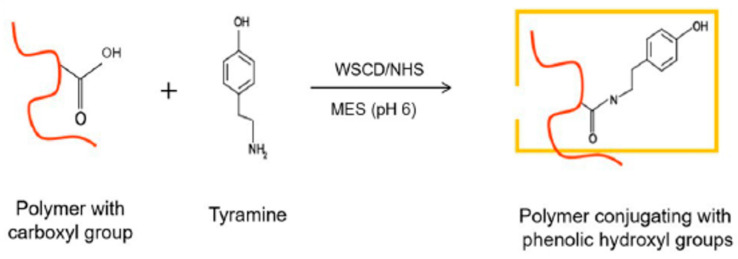
Schematic diagram of the phenolization process.

For phenolization, 5 g of alginate was dissolved in 10.65 g of MES buffer (pH 6.0) to a final concentration of 1% (*w*/*v*). The solution was left overnight to fully dissolve. After that, 3 g of tyramine hydrochloride, 0.4265 g of NHS, and 1.4185 g of water-soluble carbodiimide (WSCD) were added to start the phenolization reaction (Figure 12). This mixture was stirred at room temperature for 48 h. To end the reaction, the pH was adjusted to 8.63. The product was then precipitated using 1.5 L of acetone. The resulting solid was washed with an 80:20 ethanol–water solution until the absorbance of the supernatant at 275 nm (measured by a UV–visible spectrophotometer) was below 0.04, confirming the removal of unreacted tyramine. Finally, the sample was dried in an oven at 50 °C overnight. The successful attachment of tyramine to the alginate was confirmed using UV–visible spectroscopy (V-630 iRM Type, JASCO EUROPE, Lisses, France). The analysis verified the presence of phenolic groups in the final product.

### 3.3. FTIR

Alginate and Alg-Ph were analyzed using a Fourier transform infrared spectrophotometer (Nicolet™ iS™5, Thermo Scientific, Waltham, MA, USA) equipped with an attenuated total reflectance accessory (iD7, Thermo Scientific, Waltham, MA, USA). Spectra were collected in the range of 4000–500 cm^−1^, with a resolution of 4 cm^−1^ and 32 scans. Background was subtracted (atmospheric spectra). Notably, the peak corresponding to the carboxyl group appears around a wavelength of approximately 1600 cm^−1^. The specific steps are as follows: Take 0.5 g of sample (ensuring it covers the laser under the probe), lay it flat in the center of the disk, then pull down the locking sleeve to ensure the probe makes contact with the sample, and begin the detection. The measurement needs to be repeated three times, and the result with the fewest impurity peaks should be selected.

### 3.4. Optimization of Crosslinking System

The gelation time of solutions containing three different concentrations of the Alg-Ph in water was measured. An amount of 450 μL of the solution was poured into a 48-well plate. Subsequently, 40 μL of SPS solution was added to each well to final concentrations of 1, 2, 3, and 4 mM, and the plates were stirred at 150 rpm using a magnetic stir bar. Finally, 10 μL of ruthenium solution was added to each well while stirring was continued to achieve final concentrations of 1, 2, 3, and 4 mM. Similar to enzyme crosslinking methods, 10 uL of HRP solution was added to each well for final concentrations of 5, 10, 15, 20 U/mL, and then stirred at 150 rpm. Finally, 10 uL of H_2_O_2_ solution was added to each well while stirring was continued to achieve final concentrations of 0.1, 1, 10, and 50 mM. Gel formation was indicated when magnetic stirring was inhibited and the solution surface expanded. The effects of these parameters on gelation time were determined by varying the concentrations of SPS/ruthenium and H_2_O_2_/HRP [49].

### 3.5. Rheology Analysis

Viscosity analysis was performed using a rheometer AR-2000 (TA Instruments, New Castle, DE, USA). The instrument was equipped with a cone–plate setup, with an upper cone of 40 mm diameter and a 4° angle, and a lower cone of 40 mm diameter with a smooth cone (52 μm gap). Viscosity measurements were performed using Tektronix Instruments Rheology Advantage software (V5.7.0) [50]. Steady shear flow properties of Alg-Ph were examined at concentrations ranging from 0.5 to 2% (m/m%). After mixing with the enzyme crosslinking agent or photocrosslinking agent to a final volume of 800 μL, immediately inject the sample into the center of the platform using a pipette. Then lower the probe; the sample will naturally spread evenly on the platform. Begin the measurement and repeat three times. Cone–plate geometry was used. For the time sweep, the time was set to 1800 s, with a fixed oscillation frequency of 1 Hz and a 2% strain. All samples contained 4.0 mM SPS and 1.0 mM Ru(bpy)3 and were measured using a cone–plate system with a 52 μm gap as described above [51]. A ring of LED lights was placed around the gap and the LEDs were turned on for evaluation under visible light exposure. The system was kept in the dark for the first 120 s, with the light manually turned on at 120 s until the end of the measurement. Viscosities before and after crosslinking were compared using the Steady State Flow program with a shear rate range of 0.01 to 100 s. Viscosities before and after crosslinking were always compared at 0.01 s. Time course measurements were performed at 25 °C, at a strain range of 5–100%, and at a frequency of 1.6 Hz for 5 min. For comparison, use 1 mM H_2_O_2_ and 10 U/mL HRP enzymatic crosslink system, conducting the same process without light.

### 3.6. Texture Analysis

Texture parameters were measured using a uniaxial compression test using a Texture Analyzer TA-XT 2i (Ametek, Lloyd Instruments Ltd., Fareham, UK). This test measures the force required to compress a cylinder of scaffold between two steel plates (similar to squeezing by hand). The compression test parameters were compression plate P75; test speed 1 mm/s; target mode strain; strain 70%; trigger distance 5 mm. The height of the cylindrical sample is 0.8 cm and the radius is 1 cm. Two parameters were analyzed from the resulting stress–strain curve: hardness (H), defined by the maximum force, and elastic modulus, defined by the elastic region of the sample (the period during which the initial shape is recovered when the stress is removed). Texture testing was performed in triplicate [52].

All the significance used in this study was analyzed via ANOVA, and when significant at *p* ≤ 0.05, Fisher’s least significant difference test (LSD, *p* ≤ 0.05) was used for comparison of means. All experimental samples for significance analysis were repeated three times (crosslinking time, rheology, and texture analysis).

## 4. Conclusions and Perspective

This study successfully modified alginate using tyramine grafting, transforming them into effective hydrogel-forming materials. The degree of phenol functionalization was first quantified, confirming the efficiency and feasibility of the chemical modification steps. Next, we optimized the photocrosslinking system and identified 1 mM ruthenium and 4 mM sodium persulfate (SPS), 10 U/mL HRP, and 1 mM H_2_O_2_ as the optimal concentrations for efficient and controllable gelation. We also determined the minimum gelation concentrations for two hydrogels. Rheological analysis tracked the transition of each bioink from a liquid state to an initial gel, comparing changes in viscosity, storage modulus (G′), and loss modulus (G″) before and after crosslinking. Thixotropy and textural profiling further revealed important physical characteristics of the hydrogels, including their rigidity, elasticity, and cohesiveness. Young’s modulus testing showed how hydrogel stiffness varied with polymer and photoinitiator concentration, providing a clear comparison of mechanical performance across formulations. Scaffold fabrication using both 3D bioprinting and silicone molding demonstrated the printability and structural integrity of the materials under different processing methods. These results highlight the versatility of the two bioinks and their suitability for hydrogel-based scaffold fabrication. In future work, we will evaluate the cytocompatibility of these materials and explore their performance in intestinal tissue engineering applications. In summary, the four developed alginate-based hydrogels show strong potential for use in tissue engineering, combining tunable mechanical properties, processability, and structural support.

As a highly versatile biomaterial, alginate hydrogel functions as both a carrier for cells and therapeutic agents and as a supportive matrix in tissue engineering. It can store and gradually release growth factors and other bioactive molecules, making it valuable for regenerative applications. Over time, various crosslinking methods have been developed to fine-tune the gel’s properties for different uses. These approaches enable precise control over the hydrogel’s mechanical strength, stability, and degradation rate, supporting the creation of modified alginate systems with improved responsiveness to environmental cues and greater potential for controlled drug delivery.

## Data Availability

If the relevant data are needed, please contact the author by email.

## Supplementary Materials

The following supporting information can be downloaded at https://www.mdpi.com/article/10.3390/md24010022/s1. Figure S1: Comparison of Young’s modulus for different scaffolds.

## Abbreviations

The following abbreviations are used in this manuscript:

HRP	Horseradish Peroxidase
Alg-Ph	Phenolized alginate
SPS	Sulfonated Polysulfone
Ruth	Ruthenium
EDC	1-ethyl-(3-dimethylaminopropyl) carbodiimide
NHS	N-hydroxybutanediimide (EDC/NHS)
HA	Hyaluronic acid
GA	Gallic acid
H_2_O_2_	Hydrogen peroxide
