# Supplementary figures and images for: Tailoring the Properties of Marine-Based Alginate Hydrogels: A Comparison of Enzymatic (HRP) and Visible-Light (SPS/Ruth)-Induced Gelation
Source: Mar Drugs. 2026 Jan 2;24(1):22. doi: 10.3390/md24010022 (PMC12842925; doi:10.3390/md24010022)

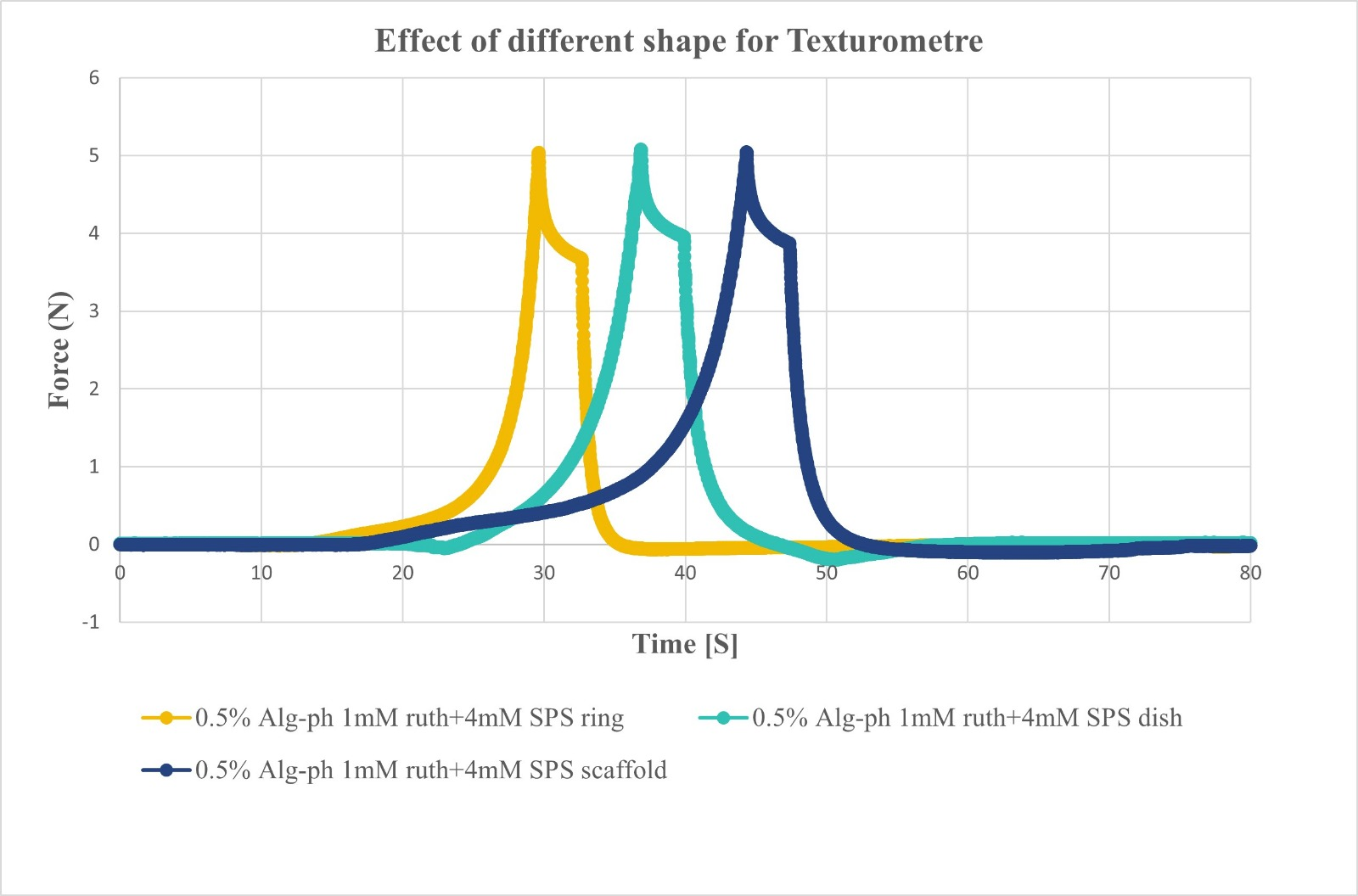

Supplement: Supplementary file 1 [file marinedrugs-24-00022-s001.zip › marinedrugs-4046686-supplementary.png]
